# Edge Length and Surface Area of a Blank: Experimental Assessment of Measures, Size Predictions and Utility

**DOI:** 10.1371/journal.pone.0133984

**Published:** 2015-09-02

**Authors:** Tamara Dogandžić, David R. Braun, Shannon P. McPherron

**Affiliations:** 1 Department of Human Evolution, Max Planck Institute for Evolutionary Anthropology, Leipzig, Germany; 2 Center for the Advanced Study of Hominid Paleobiology, Department of Anthropology, George Washington University, Washington, D.C., United States of America; University of Oxford, UNITED KINGDOM

## Abstract

Blank size and form represent one of the main sources of variation in lithic assemblages. They reflect economic properties of blanks and factors such as efficiency and use life. These properties require reliable measures of size, namely edge length and surface area. These measures, however, are not easily captured with calipers. Most attempts to quantify these features employ estimates; however, the efficacy of these estimations for measuring critical features such as blank surface area and edge length has never been properly evaluated. In addition, these parameters are even more difficult to acquire for retouched implements as their original size and hence indication of their previous utility have been lost. It has been suggested, in controlled experimental conditions, that two platform variables, platform thickness and exterior platform angle, are crucial in determining blank size and shape meaning that knappers can control the interaction between size and efficiency by selecting specific core angles and controlling where fracture is initiated. The robustness of these models has rarely been tested and confirmed in context other than controlled experiments. In this paper, we evaluate which currently employed caliper measurement methods result in the highest accuracy of size estimations of blanks, and we evaluate how platform variables can be used to indirectly infer aspects of size on retouched artifacts. Furthermore, we investigate measures of different platform management strategies that control the shape and size of artifacts. To investigate these questions, we created an experimental lithic assemblage, we digitized images to calculate 2D surface area and edge length, which are used as a point of comparison for the caliper measurements and additional analyses. The analysis of aspects of size determinations and the utility of blanks contributes to our understanding of the technological strategies of prehistoric knappers and what economic decisions they made during process of blank production.

## Introduction

Modern prehistoric lithic studies are mainly concerned with fundamental questions of how and why flintknappers produced implements of certain forms. Flake morphology, as expressed in size and shape, is one of the main aspects of variation to be investigated in lithic technological studies [1]. Depending on the research questions and perspectives, variation in blank morphology is used to infer differences in core types (morphologies) that further reveal technological traditions [2–5] or as an indirect reflection of adaptations to different environmental conditions or mobility where blank design may change as a response to certain economic strategies [6–16]. A significant component of technological organization studies is concerned with how portability and utility of blanks are affected by mobility or raw material availability in an attempt to understand how lithic technology is organized and integrated in the overall system of settlement and mobility. Within this framework a great deal of attention is paid to the efficiency of blank production and utility of blanks [7,17–20]. The concept of efficiency, recognized early on in the work of A. Leroi-Gourhan as a proxy for technological progress through time [21] can be modeled in an evolutionary framework where it potentially reflects technological decisions that facilitate meeting daily subsistence requirements. Consequently these can be considered as equivalent or at least highly correlated with genetic fitness [22].

These questions have been in focus especially when estimating the efficiency and productivity of certain core technologies [23–25] or when comparing different flaking systems [26–28]. The question of differences in efficiency of blanks produced by different technologies has been particularly important when comparing flake versus blade blanks and is repeatedly discussed in the context of the appearance of modern human behavior as blade industries have been considered an archaeological marker of modern humans and therefore an expression of ‘modern behavior’ [29]. Although a strict association between blade technologies and these cognitive rubrics has been challenged [30,31], this technology is still thought to have several advantages over standard flake production. Possibly one of the most important advantages is the increased length of the cutting edge per unit weight [28,32–35]. There is, nevertheless, an opposing view that argues for the increased economic properties of flakes compared to blades as the former can be repeatedly resharpened due to their larger initial surfaces. Arguments for the increased efficiency of standard flakes centers on the increased use-life of tools that can be resharpened multiple times. Flake width, which is usually greater than on blades, allows for multiple generations of use through retouch [26,28]. Some archeological studies have shown that radial flakes were at times preferred over narrow flakes because of their potential for resharpening [20,36]. In this case, emphasis on one particular blank form is seen as increasing their suitability for toolkits in circumstances of increased mobility.

Efficiency in lithic production can vary and can be controlled at different points in the manufacture process. Three parameters are often seen as important components of an efficient toolkit: 1) reducing the amount of raw material waste during core preparation, 2) increasing the number of end-products per core and 3) enlarging the length of the cutting edge produced. Even though the efficiency of the overall core production system and blank properties have been looked at independently [18,19,23,37,38], they are not mutually exclusive, because these production systems are often aimed at creating blanks with specific morphological features. In this paper, the focus will not be on the overall production system nor on distinctions between technologies, but rather on how size and utility properties are quantified in lithic analysis. Furthermore we will investigate how these patterns are achieved in the production of individual pieces. The question of where the utility of a tool lies can be viewed from different perspectives, depending on the behavioral circumstances that would favor certain design properties. While utility can be expressed in different values, two variables are underlined as relevant in assessing most aspects of blank utility: edge length and surface area [1,14,20,39]. When these variables are inspected relative to other measures of overall size (e.g. weight, thickness) they provide a relative measure of blank utility. Within this framework, we investigate methodological questions in lithic analysis related to these two variables. Estimating relative blank size using standard caliper measurements has thus far never been fully investigated. When original size is not preserved, such as in retouched elements, a need arises to assess their initial size properties indirectly, namely with remaining platform variables, platform size and exterior platform angle (hereafter EPA) [27,40–42]. They can, furthermore, inform us on how prehistoric knappers were manipulating these variables to control not only size but shape and consequently other properties such as the amount of usable edge or the tool’s potential for resharpening.

### Blank size estimations

In lithic analysis, morphological characteristics of blanks are inferred through descriptive assessments or, in a more precise manner, with metric attributes. Measurements are used together to assess shape and to calculate surface area and the edge length of a blank. Apart from recent advances in scanning technologies that use digitized images and 3D models to capture blank morphology or metric attributes [28,43–47], the most commonly used method to obtain linear size measurements is with digital calipers. There are, however, several ways to measure the length and width of lithic artifacts that are commonly used by lithic analysts [1,48,49]. As a need for reliability in metric measurements increases, some methods have been proposed as better reflections of the morphology of a blank [50]. Other measures have been suggested as improved estimates of edge length [32]. It is apparent that different measures give different estimates of the size and shape of the blank and the length of usable edge (the edge minus the platform). Previous attempts to evaluate how different methods of flake area calculations based on common measurement of length and width compare with more reliable measure of area that uses several observations along the perimeter of the flake [51] demonstrated consistent overestimations of surface area that ranges from 11% to 47% (Baumler 1979 as cited in [51]). This study, however, did not evaluate which method produces least errors. This is largely due to the variable and irregular morphology of flakes. It can be expected that blanks of more or less symmetrical or standardized form, such as blades, will have a more predictable relationship between shape measurements and subsequently edge length and surface area. Edge length prediction based on shape measurements of an asymmetrical or irregular blank will be more variable with higher inaccuracy. Moreover, the blank and tool size estimates measured by different researchers are unlikely to be compatible and hence difficult to compare. Here we investigate measures of size and shape based largely on caliper measures under the view that the majority of data collection still relies heavily on caliper measurements rather than scanning techniques. Considering the fact that methods may vary significantly, the first aim of this paper is to examine the accuracy of the commonly used length and width measurements to determine the error in measures of edge length and surface area and to find which of them provides the best estimate of these variables. Using an experimental assemblage, four different measurement methods are tested against the results obtained with digitized images.

### Platform as an indicator of blank size

It is widely accepted that patterns in lithic assemblages are affected by the intensity of reduction and that evaluating the extent of reduction contributes to explaining variability in lithic assemblages. This is specifically the case for retouched tool types. The Frison effect [52,53] suggests that much of the variability seen in typological categories lies in the extent of retouch. This concept contributed to a rejection of conventional typological units as discrete units, especially in Middle Paleolithic research [54–59]. Another significance of this concept lies in its potential in examining the notion of curation and repeated use of implements [14,60]. In this context, measures of tool reduction, along with other assemblage characteristics of, e.g. core reduction etc., give insights into the overall level of curation that an assemblage represents [39,61]. This has further implications in how technology is organized in specific contexts, such as increased mobility or transport patterns at certain distances to raw materials [62].

In evaluating this aspect of retouched tools, the central issue becomes reconstructing the original size of a reduced tool to accurately quantify the amount of mass removed from the blank as a result of retouch. Various measures, ratios and indices, have been devised for this purpose [46,63–68]. While most of them are focused on measuring the extent of retouch, one is specifically aimed at reconstructing the original blank size as compared to the discarded size [65]. The problem of size reconstruction is directly related to the question of how knappers control the detachment of a flake and, more importantly, its size. Several experimental studies have examined flake variation, mainly dimensional attributes, resulting from variables controlled by the knapper ([40,42]–Fig 3x). These experiments, and many subsequent studies [27,69,70], indicate that the size of a flake is primarily determined by platform thickness and EPA. The high predictive power of platform size in assessing flake size was used to infer original size of a blank before some amount of it has been removed by retouch. In the study of reduction patterns and their effects on typology, Dibble [64] used the ratio of the remaining surface area to platform area as a relative measure of tool reduction.

Some researchers have expressed concern over how well this ratio works to identify the degree of reduction in unifacial tools [71,72]. Many of these concerns revolve around the question of platform thickness’ ability to predict size despite the positive correlation between the two. Emphasis on curation in lithic studies has inspired further search for the best estimate of reduction. The predictive power of platform area stimulated novel techniques for precisely calculating its area by scanning to improve its reliability as a predictor for blank size [44,46,73].

Another line of research that contributes to this question is looking at flake size variation as a factor of not only platform variables, but EPA as well. These two variables are crucial in determining the size of the blank [40–42,70]. If we consider that this relationship is established and reliable, then size predictions should be more usable with these platform variables. The next step is to evaluate the robustness of these model relationships derived from controlled experiments on knapped assemblages. Some test studies on archaeological or experimental data were previously made, though with less precise measures of edge length and surface area [69,70,74]. In this study we used surface area and edge measurements derived from digital images to test these relationships. In this context, we will first investigate the possibilities of estimating original blank size and how well platform variables control for the size of the blanks.

### Platform as an indicator of blank shape

The concept of efficiency has been a major topic in the studies of lithic organization [1,7,18,23,35]. For instance, certain circumstances, such as mobility [20] or raw material constraints [19] may have favored modifications in production strategies towards higher efficiency or longer use-lives. Most studies that focus on the economy of lithic production stress use life and curation in retouched elements [7,15,59,63,68,75,76], though the importance of the unretouched assemblage and its role in the economy of lithic reduction strategies is equally relevant [60,77,78]. While there are different concepts of flake utility [14,20], two main currencies appeared to be especially important: 1) efficiency index as a measure of usable cutting edge per unit of mass and 2) retouch potential, i.e. the possibility of further modifying the edge that would extend a tool’s use-life.

Tool efficiency has been a focus in recent studies [18,19,32,79], though its importance in the studies of technology and evolution was recognized earlier [35]. Leroi-Gourhan considered the gradual maximization of a tool’s cutting edge against its diminishing mass during Paleolithic times as a step towards efficiency that marks technological progress [21]. Varied blank morphologies can be a solution for situations where higher efficiency is required [1,14,20,80,81]. Smaller blanks have larger efficiency for the same unit of weight, though larger blanks are known to have been transported larger distances [39,82,83].

Making a balance between usable edge and transport weight is not the only blank requirement that foragers may have sought. While foragers would, especially in higher mobility circumstances, tend to lower the transport costs of portable implements, another approach is to transport larger implements with a greater potential for edge renewal. This said, a blank’s retouch potential is often taken as a proxy for tool’s longevity [7,65]. A long edge is one possible prerequisite, but flakes also need to have a significant volume, and blanks of a specific form, thick and asymmetrical in section, have been suggested for this purpose [84,85]. Another, and quite different, morphological trait that increases the resharpening capacity of a blank is larger surface areas relative to thickness. This blank morphology is often associated with low edge angles for repeated resharpening events [20,24,85,86].

Experimental studies showed that knappers used certain rules to achieve specific blank shapes [40,87]. It has been suggested that the knapper can control both blank size and shape by manipulating platform depth and EPA [41]. If the variability of morphological ratios can be controlled by these platform attributes, then one can look at strategies employed in technological processes aimed at acquiring blanks of specific properties. Previous work [27] has suggested that increasing the EPA will have an impact on both of these currencies. Simultaneously decreasing the thickness will reduce the weight, which is seen as an optimal efficiency strategy [16]. When these models are applied in an archaeological context some patterns seem to emerge [69]. In our analysis these relationships are examined in an experimental assemblage where platform variables are not controlled. The platform variables vary presumably as much as they do in some archaeological assemblages. As a result this assemblage offers another opportunity to evaluate the strength of the models governing blank production.

## Materials and Methods

**Fig 1:**
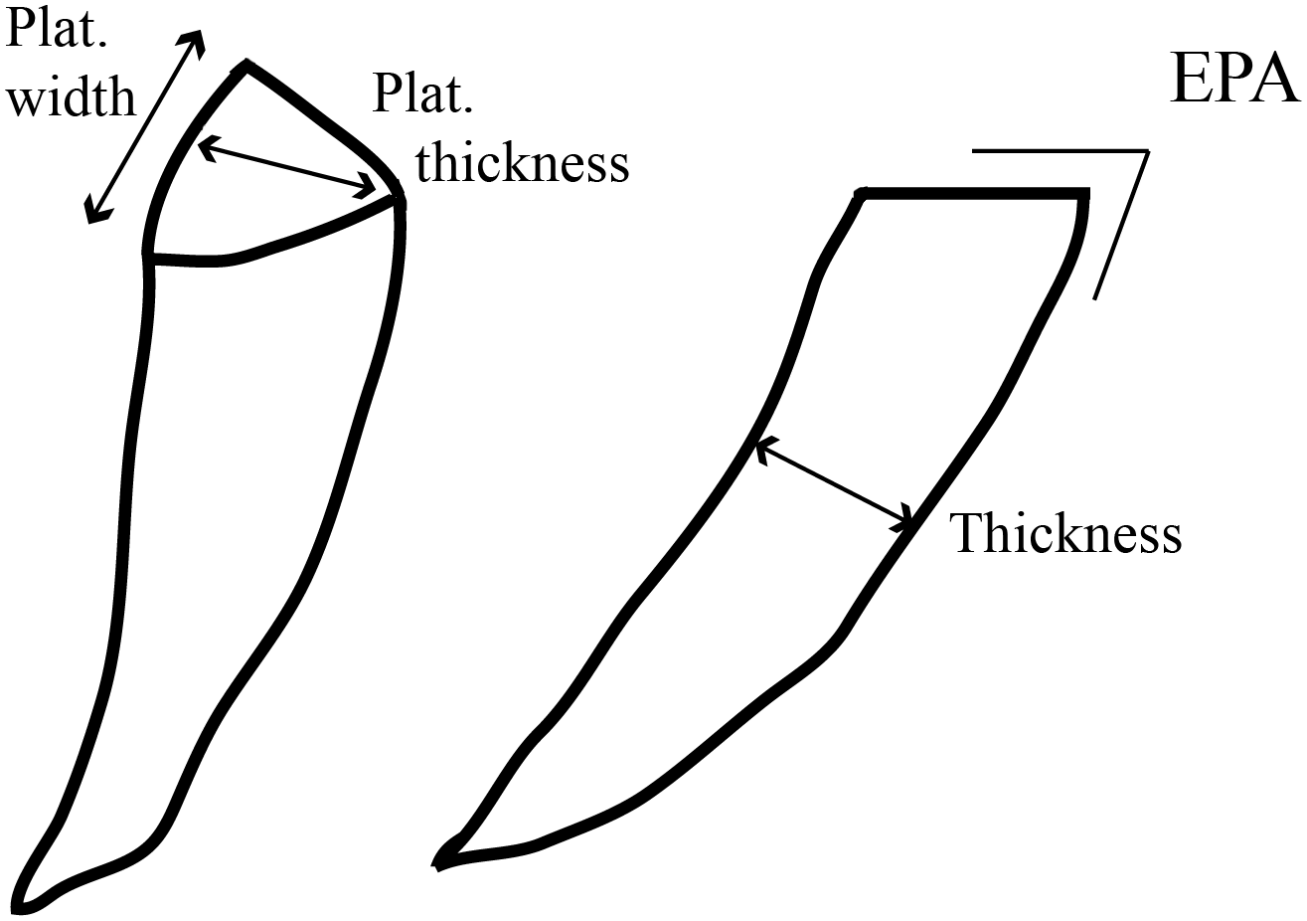
Platform variables considered in this study.

In this study we used an experimental lithic assemblage produced with freehand, hard hammer percussion. Artifacts were made on two different raw materials: flint, derived from the Bergerac region of France, and hornfels (shale that had undergone contact metamorphism) collected from the Karoo region of South Africa [88]. Bergerac flint is of good quality and comes in larger nodules of less regular shape. The hornfels nodules in this study, on the other hand, are smaller in size and were found in rounded nodules. The blanks produced vary in terms of morphology and technology as there was no preference for either during the flintknapping. The goal was to produce a variety of flakes. Some of them resemble Levallois blanks while some have a morphology that conforms to the definition of a blade. They also vary in size, shape, and the amount of cortex. The sample size varied for different parts of the study (Table 1). For the analysis that requires exterior platform angle and platform size, the sample is smaller as some pieces had cortical and curvy platforms and/or dorsal surfaces that made measuring EPA unreliable. For each artifact, we measured platform width and thickness using digital calipers to .1 mm and measured weight on a digital scale to .1 gr. EPA was measured with a goniometer to the nearest degree, and it was taken as an angle between the platform surface and dorsal surface area, along the axis of flaking (Fig 1).

**Table 1:** Sample size and summary statistics on experimental assemblage.

	Flint	Hornfels	Total
**Metric estimates**	109	126	235
**Size predictions**	99	71	170
**Length**	M = 45.74	M = 31.8	M = 38.26
	SD = 14.35	SD = 9.44	SD = 13.83
	range = 21.87–75.85	range = 17.64–69.9	range = 17.64–75.85
**Width**	M = 38.45	M = 29.76	M = 33.79
	SD = 17.82	SD = 10.21	SD = 14.87
	range = 12.29–116.2	range = 12.03–62.78	range = 12.03–116.2
**Elongation**	M = 1.37	M = 1.17	M = 1.27
	SD = .62	SD = .48	SD = .56
	range = .44–3.32	range = .5–2.61	range = .44–3.32
**Skew angle**	M = 21.36	M = 23.03	M = 22.25
	SD = 16	SD = 16.5	SD = 16.25
	range = .08–64.1	range = .54–65.6	range = .08–65.6

**Fig 2:**
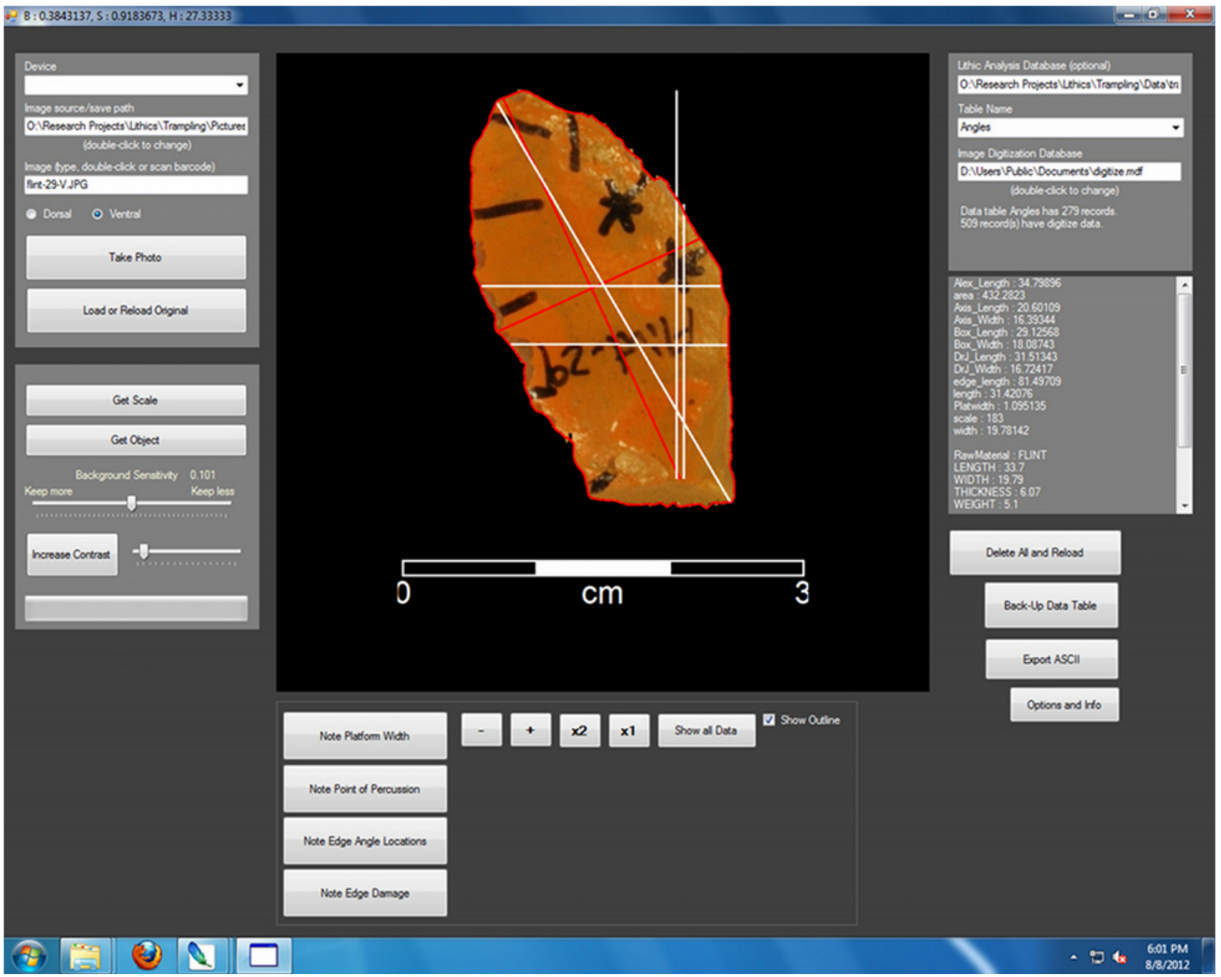
Screen shot from the program used to digitize artifacts. It takes artifact photos, extracts the scale, artifact outline, takes various measurements of length, width and calculates the edge length and surface area (without the platform).

We used digital images of artifacts to calculate the edge length and blank surface area. First, both the ventral and dorsal side of each flake was photographed with a digital camera with a centimeter scale near the platform. Flakes were placed in a container of salt so that they could be easily made flat and also to insure that the scale was captured at the same focal depth as the perimeter of the flake [47]. Flakes were aligned with the axis of percussion. Digital images were then loaded into custom written software [88]. The program counted the number of pixels per centimeter in the scale, and after automatically removing the background around the artifact, the software produced a set of XY points to represent the artifact outline (Fig 2). On the ventral side image, we noted the point of percussion as well as the limits of the platform. Length and width measurements according to each measurement method were automatically calculated using the edge outline and the point of percussion. Edge length and surface area were calculated without the platform length and area.

**Fig 3:**
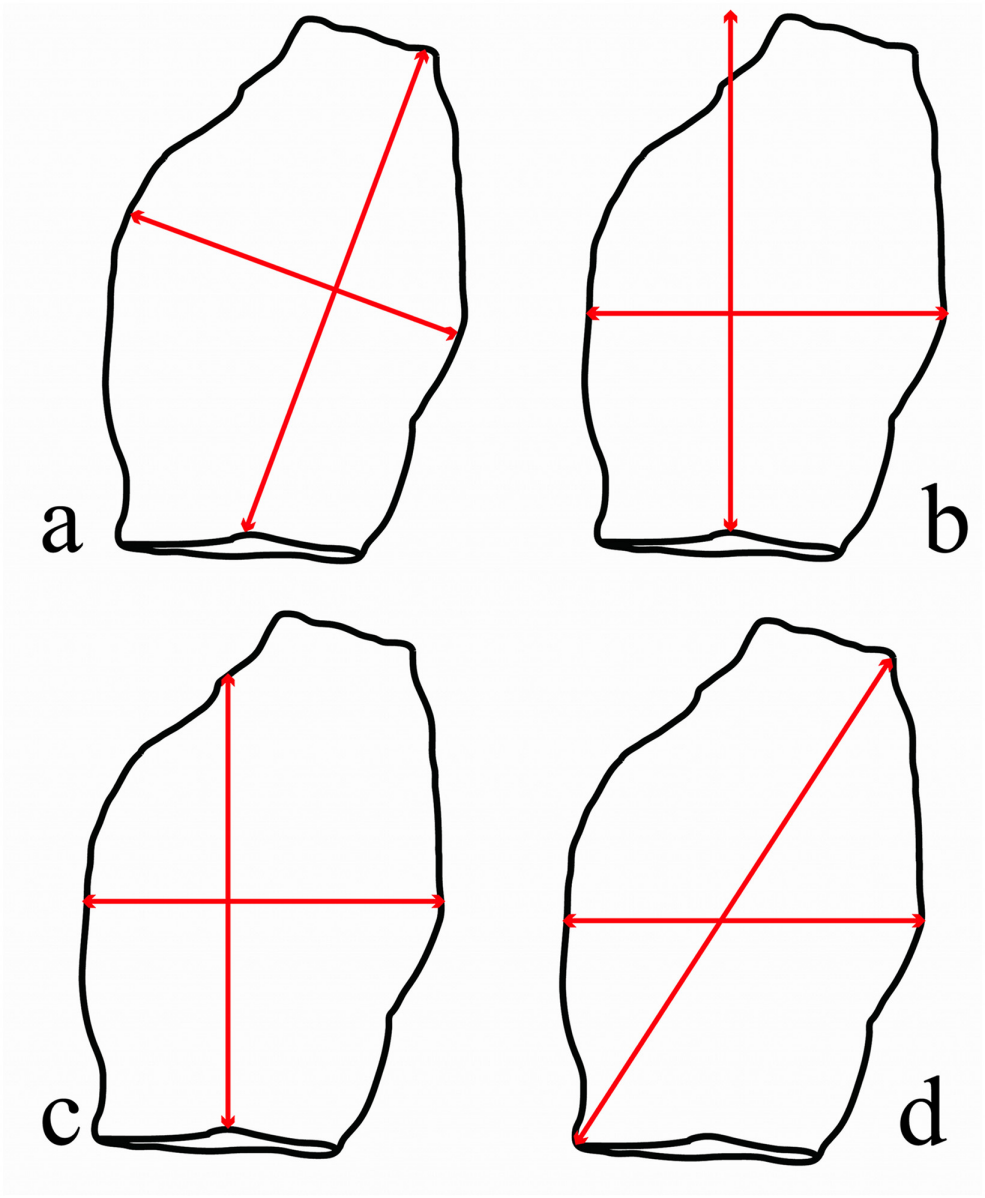
Different measurement methods most often used by lithic analysts. a) Long axis method—Length is recorded from the point of percussion to the farthest distal end of the blank. Width is measured at the midpoint of the length axis and perpendicular to it [92]. b) Box method: Length is the maximum distance from the point of percussion to the distal end, following the axis of percussion (perpendicular to the striking platform width). Width is taken perpendicular to the length, at the mid-point of the length. c) Axial method–Length is a distance represented by the straight line from the point of percussion to the distal end, following the axis of percussion. Width is taken at the midpoint of the length, perpendicular to it. d) Longest measure method for edge length: Maximal length is taken as maximum distance across the flake and not tied to the point of percussion. Length is axial length and width is not the maximum perpendicular to the length as suggested by [32], but same as Box width.

One of the most commonly used measurement system is following the axis of percussion of the artifact [89] (Fig 3c). For symmetrical pieces this method is a good estimate of size and surface area, but when the axis of flaking and axis of symmetry diverge from each other, this method may underestimate the overall size of the piece. To overcome this disadvantage and to preserve information on flake shape, Jelinek, following Leach [90], used a different method (Fig 3a, named Long axis method) in his study of the Tabun assemblage. Jelinek’s measure of length is taken from the point of percussion to the farthest point on the edge of the flake. The assumption is that this measure is a better reflection of the true outline than the standard axial measurement scheme [39,50,91,92]. Taking the maximum length and width measurements (Fig 3b), the so-called box method [49], is also employed by many researchers (e.g. [93,94]). Here we use a modified method where width is not maximal, but at the mid-point of the length measurement (Fig 3b). Finally, dissatisfied by the usual formula for calculating edge length, 2*Length+Width, that often results in inaccurate estimates for the pieces whose maximal dimension is not its axial length, Mackay’s [32] study of changes in flake efficiency in South African Middle Stone Age assemblages presented a new method designed to be a better estimate of flake edge length. It uses the longest measure instead of length (Fig 3d). Using linear measurements obtained by the software [88], edge length and surface area of every artifact were then calculated according to each measurement system. Surface area is calculated as a product of length and width, edge length as a width plus two times the length, and the Mackay edge length as a sum of length, maximum length and width. Statistical analysis was done in R [95].

## Results and Discussion

### Blank size estimates: length, edge length and surface area

#### Size Measures with Calipers

Assemblage comparisons often rely on similarities and differences in one or more absolute measures of artifacts, such as length and width. Depending on the flake morphology, the choice of measurement method could produce variable results. For example, lengths taken with three different measurement systems (box, long axis and axis) for 241 blanks used in this study are statistically different (Kruskal-Wallis, χ^2^(2) = 21.49, *p* < .01), with the largest difference being between the long axis method and axial measurement techniques (post-hoc Kruskal-Nemenyi test, *p* < .01) and box and axial (post-hoc Kruskal-Nemenyi, *p* < .05). Obviously, difficulties can arise when assemblage comparisons based on different metric systems are interpreted.

**Fig 4:**
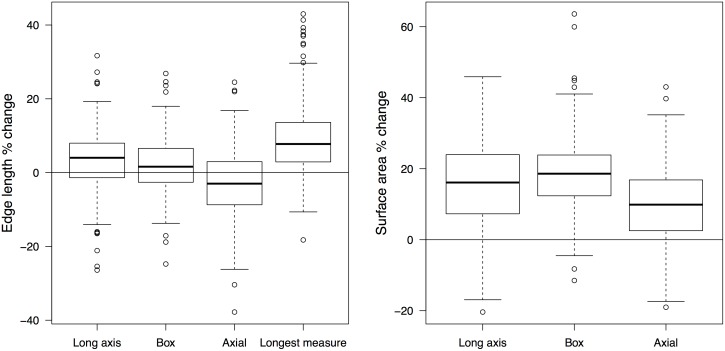
Average errors of edge length (a) and surface area (b) by different measurement methods.

Many research questions concerning lithic technologies, especially ones that tackle aspects of technological economization, are, nevertheless, concerned with the length of the usable edge and the area of a blank, rather than length or width of a blank alone. For that purpose, estimations are made from measures of lengths and widths. Here we analyzed how edge length and surface area calculated using different caliper measurement methods compared to ones made from digitized images. In method comparisons for surface area estimates, Mackay measurement method was not included, as it was not aimed at obtaining this value, and, as it uses the maximum measurement, would consistently overestimate the area. Therefore we only compared three other measurement methods. While regression analysis indicates that all methods give good prediction for the edge length (all *r* ^2^ >.92) and surface area (*r* ^2^>.95), Kruskal-Wallis tests showed that means and variation between metric systems significantly differ for edge length (χ^2^(4) = 18.83, *p* < .01) and surface area estimates (χ^2^(3) = 9.77, *p* < .05)… Pairwise comparisons reveal that the significant difference is between digitally obtained edge length and the estimate with longest measure method and axial method (Kruskal-Nemenyi, *p <* .01). The largest difference with the surface area measurements is with the box method (Kruskal-Nemenyi, *p* < .05). An examination of the degree of error of each system compared to digitized length or area gives an insight into how reliable each estimate is. As shown on Table 2, measurement errors can be large, with some percentage errors up to 30–40% for edge length and 60% for surface area and the measurements can equally over- or underestimate the value (Fig 4). What is required in any analysis of lithic metrics is a measure that is most accurate, i.e. shows lowest mean percentage error, and has the highest precision, showing the lowest variation in this error. As evidenced by the data on Table 2, length and width measurements taken with the box method give the best estimation of the length of the edge. Similar results are obtained for the Jelinek’s method as well, while the other two to a large extent over- or underestimate this value. Surface area calculations, on the other hand, are subject to larger errors and consistent overestimates. The axial method lies closer to the digitized image values for blank area, most probably because it represents the most conservative measurement method and it usually excludes, mainly for irregular pieces, some parts of the blank surface.

**Table 2:** Summary statistics of errors in edge length and surface error estimations with different measurement systems.

**Edge Length: % error**	**Long axis**	**Box**	**Axial**	**Longest measure**
**Mean**	3.24	1.9	-3.36	9
**SD**	8.32	7.36	9.24	9.92
**Range**	-26.41–31.7	-24.77–26.86	-37.8–24.51	-18.24–43
**Surface Area: % error**	**Jelinek**	**Box**	**Axis**	
**Mean**	15.27	18.68	9.75	
**SD**	12.61	10.56	10.7	
**Range**	-20.38–45.9	-11.49–63.58	-19.02–43.03	

It is important to understand why some of these estimates are prone to such large errors. Linear measurements view blank form as an idealized, regular quadrilinear geometric shape, which is rarely the case. It is apparent that symmetrical blanks would have a predictable surface area and perimeter, clearly determined and easily calculated by length and width. Moreover, length and width measurements taken with different methods would largely overlap. For irregular blanks, however, this relationship is not so straightforward. One of the variables considered to be an indicator of the blank morphology is angle of skew [92]. Here we define it as the angle between the axis of percussion and the line between the point of percussion and most distal point of a blank (equivalent to length taken according to Jelinek’s measurement), given that the axis of percussion has an angle of 0°.

For extreme misestimates of edge length and surface area, i.e. the outliers in estimation errors, morphological patterning is observed: their average angle of skew is high, 40.4°, and length to width ratio, another measure of blank shape, is usually less than 1 (on average .73). These outliers indicate that morphology does play a role in estimation errors. Moreover, flint specimens showing extreme estimate errors are among the largest in the assemblage, with a maximum length of 72.1mm, suggesting that the estimations are less reliable for large pieces with higher skew angles. Correlation analysis shows that deviation from the expected value of edge length for long axis (*r*_*s*_ = .048, *p* = .46) and box (*r*_*s*_ = -.06, *p* = .38) method is not significantly correlated with the skew angle, while for axial (*r*_*s*_ = .13, *p* = .04) and longest measure (*r*_*s*_ = .14, *p* = .03) positive correlation is demonstrated. In other words, box and long axis methods are less susceptible to variations in blank morphology. The previous study that aimed at finding a better estimation of edge length [32] showed a strong correlation between this method and digitized edge length which was confirmed in our sample as well (*r* ^2^ = .95, *p* < .01). Mackay, however, did not test this new measure against others. This method had the largest number of outliers, and the largest errors as well as substantial influence from skew angle and blank morphology. This measure does not prove itself as the most reliable of the measurement systems for edge length estimates.

In predicting the surface area, errors for box method are positively, though weakly (*r* = .13, *p* = .047) correlated with angle of skew, errors of the long axis method negatively (*r* = -.28, *p* < .01), while the reliability of the axial method does not depend on the irregularity of the flake (*r* = -.1, *p* = .095). In other words, the more irregular the flake is, the box method shows slight increase in error in predicting area, and conversely, the long axis method gives more reliable results for less symmetric blank shapes. Interestingly, Jelinek’s method was initially aimed at capturing the morphology of the blank [50,92] yet the present study suggests that this measure exhibits less error in predicting the surface area for irregular pieces.

#### Indirect Size and Shape Estimates with Platform Variables

What lithic analysts are more often concerned with is inferring original blank size when the question of interest is how much of the original tool was lost through retouch modifications. For this purpose, only measures not affected by retouch can be used to reconstruct the original blank size. In his work on reduction patterns among tool types in the Middle Paleolithic, Dibble [64,65] used a ratio of surface area to platform area as an assessment of the amount of reduction a tool underwent. The underlying assumption is that platform area and surface area are linearly related and, therefore, display a constant ratio. Blanks with lower than expected ratios indicate tools whose surface areas have been more reduced. This measure is relative and can be applied on an assemblage-wide scale, looking at relative reduction between tools. In Dibble’s original study [65] the ratio was compared between different tool types to show that as one moves from one tool type to another this ratio changes. As controlled experiments demonstrated the independent effect of platform depth and EPA on flake variation [27,41,69], they should be integrated in the prediction of original size. Moreover, in many previous studies it has been emphasized that predictions should consider and include as many variables as possible [46,96]. It is important to focus on the variables that are preserved despite retouch. Blank thickness, which in many cases remains preserved even after retouch [96], is a variable that can be incorporated into these estimations of initial flake size. We tested the possibilities of predicting blank size with multiple regression analysis, with platform thickness, platform width, EPA and blank thickness as independent variables. As retouching a tool’s edge reduces its size in two dimensions, weight (as a proxy for mass) and surface area, both are considered as dependent size variables.

**Fig 5:**
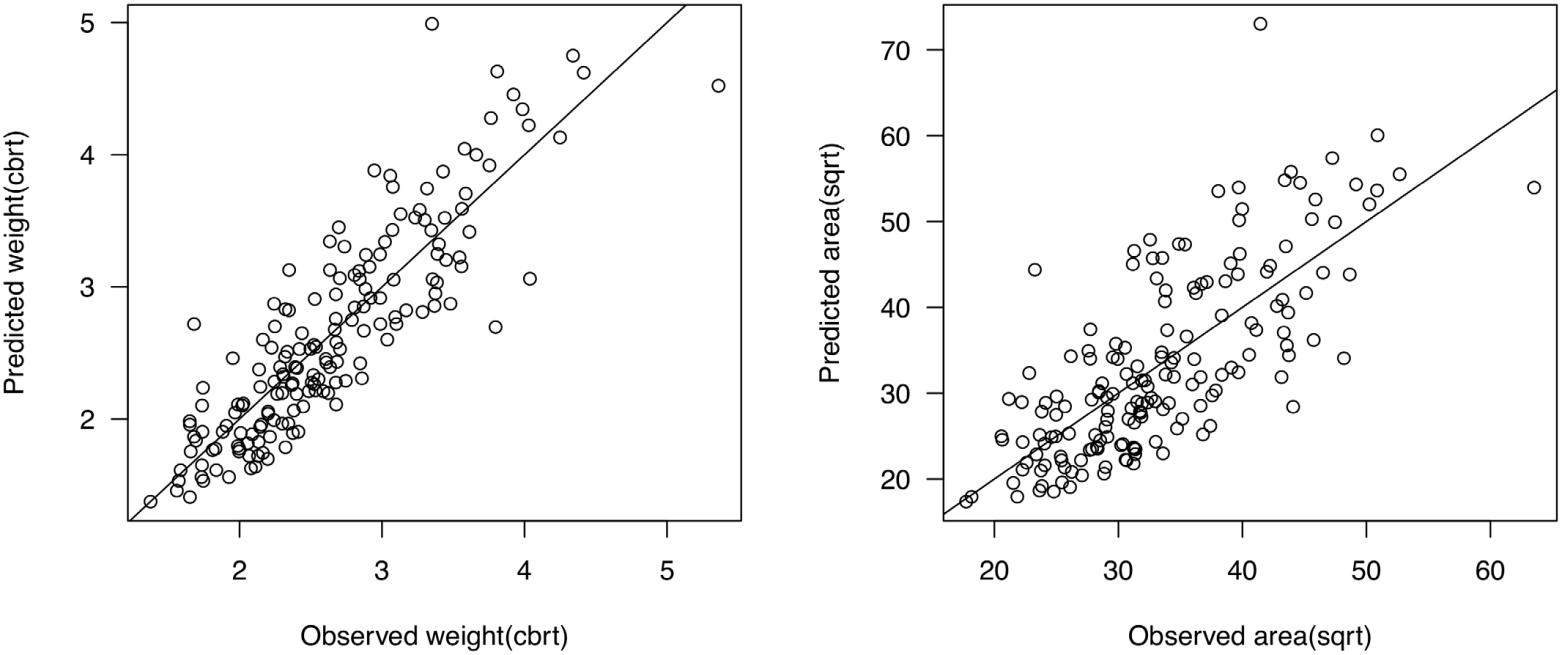
Plots of observed vs. predicted values of weight and surface area resulting from multiple regression.

We performed multiple linear regression with platform width, thickness and EPA together and found a significant but weak correlation with mass, with *r* ^2^ = .49, *F*(3,166) = 54.69, (in order to achieve linearity for the regression all models include cube root of weight and square root of platform area and dorsal surface area). With blank thickness included in the model, the coefficient of regression significantly increases (*r* ^2^ = .75, *F*(4,165) = 128.1, *p* < .01), explaining 75% of the variation by independent variables. ANOVA comparison of models with and without blank thickness shows that thickness adds to the result of linear regression (*F*(2,166) = 175.68, *p* < .01). Using these three predictor variables, regression analysis gives significant results for predicting surface area as well (*r* ^2^ = .54, *F*(4,165) = 50.47, *p* < .01), though the regression coefficient is not as strong as when predicting mass. A stronger correlation exists between the predicted and observed values for the regression model for weight than for the surface area (Fig 5). Coefficients of regressions for predicting either of the size variables are significant but not strong for either. Moreover, there are differences in the regressions for the two raw material groups, with hornfels assemblages showing a slightly higher correlation coefficient (*r* ^2^ = .8, *F*(4,66) = 69.79, *p* = < .01) than flint (*r* ^2^ = .73, *F*(4,94) = 66.65, *p* < .01). The same is true for surface area (hornfels: *r* ^2^ = .6, *F*(4,66) = 27.3, *p* < .01; flint: *r* ^2^ = .47, *F*(4,94) = 22.93, *p* < .01). Plots of observed versus predicted values from regression models for weight and area (Fig 5) show that the models may not be accurate enough, which can lead to higher uncertainties in using the equation for interpretation of tool reduction on an artifact by artifact basis.

In certain flake forms, surface area decreases more rapidly with retouch than mass (i.e. flakes with high edge length relative to mass). In these instances it would be advantageous to be able to predict flake surface area with high accuracy (to be able to distinguish tool forms with slight retouch versus extensive retouch even though mass may not change dramatically). Regrettably, surface area prediction from multiple linear regression explains less variation in surface area than mass [74]. Initial-/terminal size comparisons [46,71] can be used as a ratio of the discarded size (mass or surface area) to the size estimated by the platform variables. These ratios can act as an estimate for the amount of reduction in an assemblage. This ratio is, in the case of no reduction, expected to be equal to one. The ratio of weight predicted by multiple regression to the original weight measured in our assemblages is on average equal to one (Table 3). While these mean values are encouraging, the random error in size predictions on an individual piece by piece basis, which may be the result of measurement error, is an obstacle in reconstructing reduction intensity since prediction errors can be large and unable to reveal reduction patterns [96]. The other possibility is that the over- and underestimations would average out across the assemblage and assemblage based estimation of reduction would remain fairly accurate.

**Table 3:** Summary statistics of ratio of observed to predicted weight and surface area for flint and hornfels.

**Observed:Predicted Weight**	**Flint**	**Hornfels**
Mean	1	1
SD	.15	.1
Range	.74–1.47	.77–1.36
**Observed:Predicted Surface Area**		
Mean	1	1
SD	.21	.15
Range	.68–1.7	.75–1.46

One line of evidence in examining the sources of error in predictions can come from predictor variables. The LMG method for analysis of relative importance of predictor variables in multiple regression [97] reveals that thickness of a blank plays the most significant role in regression for all measures of size, contributing to 57% of r^2^ value for weight, 45% for surface area and 52% for edge length. The relative importance of platform thickness in predicting weight and surface area is 17% and 12% respectively and only 8% for edge length, platform width 20% for weight, 28% for area and 15% for edge length, while EPA has only 5% contribution to weight and 13% for surface area and reaches 25% for edge length predictions. Thicker platforms mainly contribute to an increase in blank thickness and larger bulbs resulting in higher blank weights [27]. Changes to EPA, however, affect weight at a slower rate than surface area or edge length. One possible influence EPA has on weight predictions may be the effect EPA has in producing relatively smaller bulbs of percussion. Flakes with relatively smaller bulbs have lower mass values [69]. In addition, the reduced effect of EPA on weight estimations is likely the result of the inability to measure EPA with high precision. Nevertheless, as we used response variables obtained with digitized images in order to reduce the error, we may consider that the error probably lies in the more subjective measures of predictor variables, e.g. EPA. Multiple regression beta values may predict some variables with greater confidence because of the effect on its higher measurement reliability. Non-reliable data can mask the relevance of predictors compared to more reliable ones. As for the platform size, a solution to these problems can be found in higher accuracy measures of platform variables [44,46]. As for the EPA, a solution is still sought.

**Fig 6:**
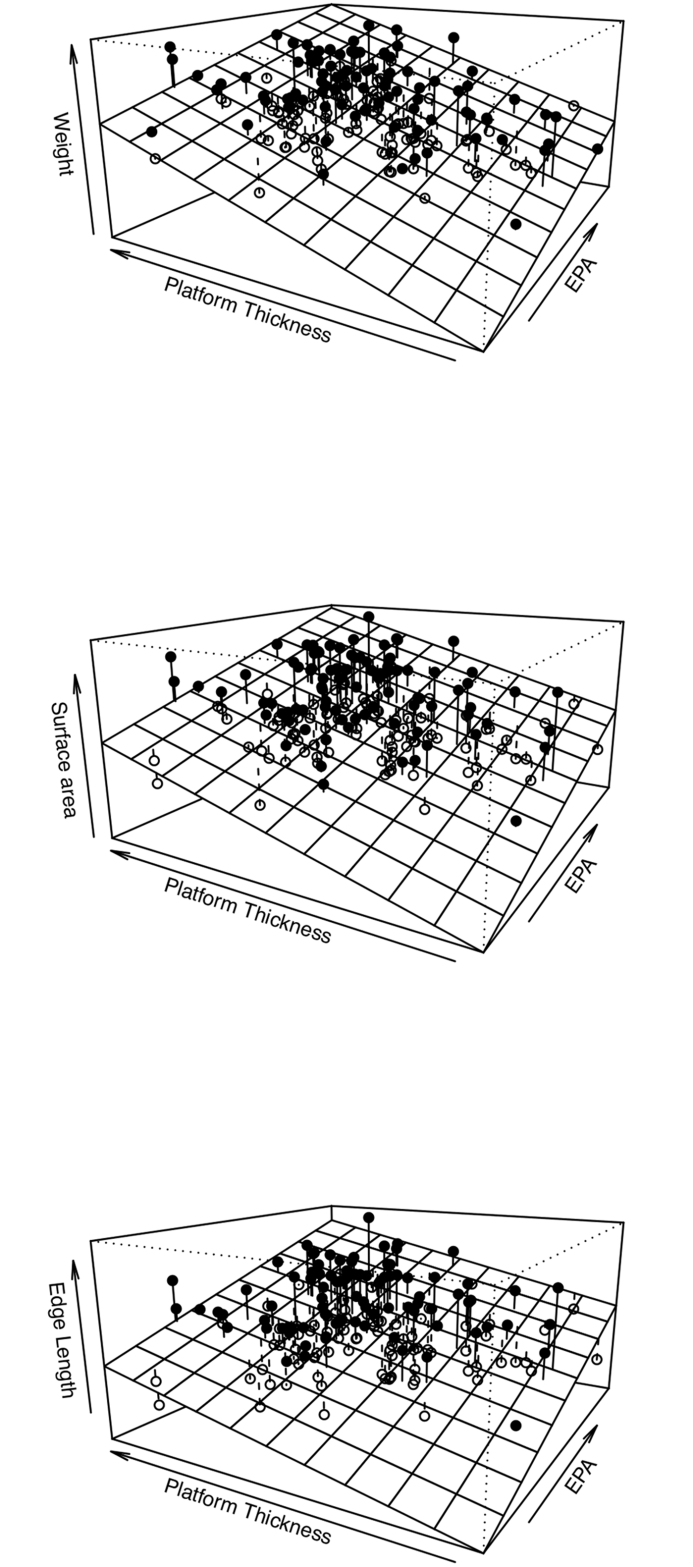
3d plots of multiple regression results with platform thickness and EPA as predictors of different size variables.

To sidestep the errors involved in direct size reconstruction on an individual artifact basis, multiple regression can be used to understand causes of variation in dependent variables and the ways predictors contribute to the overall model. Indirect insight into size and shape variation of the blank variables comes from the variables that are directly under control of the knapper, namely platform characteristics. Their power is to inform not only on the size of the blank, but also on potential tendencies in the production of blanks of different morphologies. Previous work demonstrated that both increases in platform thickness and EPA will increase flake weight or surface area. In our experimental assemblage increases in size reflects the combined effect of these platform variables (Fig 6).

Given that increasing blank area and edge length while reducing weight is a major factor when assessing the efficiency of a blank production strategy, it is clear that EPA, with a greater influence on regression models for area and edge length, has a crucial role in producing efficient blanks. Blank production strategies that increase EPA and simultaneously decrease platform area produce the most efficient blanks. The resultant blanks have shapes whereby size is optimized per unit of weight [27,42]. Efficiency, expressed as an amount of usable edge per unit weight can be obtained by changing the ratio of length to width or enlarging the blank area relative to thickness [69]. In our experimental assemblage, blanks were not produced following a specific technology (blade vs. flake production), therefor we tested the correlation between blank morphology, i.e. length to width ratio, and efficiency. There is a significant increase in efficiency with blanks that have a higher length to width ratio (*r*_*s*_ = .4, *p* < .01) which is in agreement with previous work [28,30,69]. Allometry influences this relationship as well. As blade length increases, width and thickness, and consequently mass, increase at a slower rate. As a result the overall weight of the blade increases as with increases in length [80]. The other strategy of increasing efficiency involves producing blanks with larger areas relative to their thickness. It has been suggested that in choosing either of these strategies, a flintknapper can either decrease platform depth or increase EPA [69]. The experimental data from this study do not clearly support this pattern. While EPA is positively correlated (*r*_*s*_ = .38, *p <* .01) in hornfels and platform thickness negatively correlated with the efficiency index (*r*_*s*_ = -.44, *p <* .01) in the entire assemblage, their relationships with morphology ratios are not so straightforward. EPA correlates with the elongation index only in the hornfels assemblage (*r*_*s*_ = -.26, *p* = .02), and it shows no correlation with the surface area to thickness ratio. Platform thickness is negatively correlated with the elongation ratio (*r*_*s*_ = -.25 *p* = .01) and the surface area to thickness (*r*_*s*_ = -.3, *p <* .01) only in the flint assemblage, while in hornfels correlation exists, but is not significant (*r*_*s*_ = .21, *p* = .07). While EPA and platform depth contribute to flake shape, there is still unexplained variability of blank morphology that can potentially be attributed to other factors, e.g. technology of blank production. The weak relationship of platform variables with blank morphology is possibly due to the fact that variables in experimental dataset are not held constant as in controlled experiments, where these relationships have been established. Moreover, some variation in blank morphology can be explained by variables that cannot be observed, such as angle of blow [98].

These two efficiency strategies (longer vs. large and thin blanks) would produce blanks of different morphologies. While both are beneficial in efficiency terms, they may diverge in their utility properties. Prehistoric knappers at times may have focused on producing blanks that have a higher potential for retouch. Although thick flakes may have been optimized for multiple episodes of retouch [84], the increased flake area could be achieved by producing blanks with larger areas and reduced thickness. Elongated blanks rarely exhibit this morphology [28,80]. Discrimination between these two blank forms can be informative when looking into questions of whether production was aimed for certain blank forms and their specific economic properties and the means with which they were achieved. Some studies point to the relevance of platform width that should be considered not only in prediction equations but in managing blank form as well [41,99]. In our dataset, the platform width to platform thickness ratio is positively correlated with blank area to thickness ratio (*r*_*s*_ = .32, *p* < .01) and negatively correlated with elongation ratio (*r*_*s*_ = -.21, *p =* .01). This leads us to the idea that, aside from platform depth and EPA, some morphological variation in blanks is directed by the shape of the platform [41].

## Conclusions

This study considers several aspects of blank size and shape. Studies on metric estimates are essential in issues of how much errors we have in our data and difficulties in comparing assemblages with different measurement systems. Our dataset demonstrated that estimates of flake edge length and surface areas are prone to, sometimes large, errors. Any analysis based on comparisons of not only length and width measurements, but also edge length and surface area, either within an assemblage or between assemblages recorded with different methods, will be problematic. Here we have provided comparisons of different methods that highlight when some measurement techniques are more suitable than others. The suitability of a measurement technique is highly dependent on the variable investigated and blank morphology. For the best estimates of edge length, box and long axis method revealed similar and highly reliable results. These techniques are least dependent on the irregular morphology of blanks. Surface area estimates are best obtained using axial measures, though the box and the long axis methods exhibit reasonably good results, while the latter performs well for more irregular blank shapes. As a downside, the long axis measure for considerably skewed artifacts can result in a false impression of laminarity. The long axis measure of length will always be very close to the maximum dimension, while width will rarely be at the maximum width of the piece. Given the degree of inaccuracy of some measurement systems in either or both edge length and blank area estimation, the selection of measurement system employed in lithic analysis should be driven by the research question and the morphological variability of the archaeological assemblage.

An alternative way to assess size and shape properties in blanks is estimating the size variation with the help of platform variables. These platform variables indicate how the size and shape of a blank are achieved. This is specifically aimed at reconstructing the size of modified and incomplete blanks. One way of assessing the amount of mass or surface area missing, is to predict original size and compare it with the measured value from the discarded artifact. Using multiple regression with predictor variables that are preserved in tools after they have been retouched indicates that mass is predicted with greater accuracy than surface area. The error produced by the equation or ratios aimed at reconstructing the original weight make it difficult to precisely and accurately measure size. Surface area would be more extensively affected by retouch as it reduces faster than weight. This may be because the weight removed by the retouch is not substantial. It is, nevertheless, not predicted by platform variables as well as mass is. One source of error may be in imprecise measures of predictor variables. There are still difficulties in overcoming this problem. First, there are different opinions on how the EPA should be measured [42,99] and any error in validity of this measure may lower the predictive power of this variable when compared to controlled experimental conditions. This said, there is certainly more room for investigating how different EPA management techniques (beveling, abrasion, etc.) affect blank size and morphology, and consequently, how to measure EPA in lithic analysis. Second, obtaining digitized images of platform area, though tedious and/or expensive task to perform on very large assemblages, can be feasible in some cases and has been shown to improve the estimations of size for individual pieces [19,46]. Since blank size is seen as an outcome of variables other than platform size and angle, such as angle of blow, type of hammer, size of raw material etc. [98], misestimations of size can be due to these other variables or their interactions. These factors most likely account for the variance not explained by our independent variables. Given our results, nevertheless, regression models can still provide a relative measure of mass or area loss, rather than as an equation for realizing exact size values on individual pieces.

If prediction on an individual basis is not possible and scanned platform or more reliable EPA measures are not available, examining tendencies in how a certain morphology is obtained with regard to variables that can be controlled for (i.e. platform size and EPA) can be used as an additional inference to blank production tendencies [100]. A question we are interested in is how much morphology and efficiency properties are influenced by platform variables directly manipulated by the knapper. Previous research demonstrated how changing EPA and platform thickness produces blanks of different size, morphologies and utility characteristics. Considering that there are different ways to increase size values, there may have been different strategies to increase the properties of blanks, such as enlarging the length of usable edge while lowering the mass, i.e. reducing transport costs, or extending the use-life of a tool that could be resharpened multiple times. According to the results of controlled experiments both of these measures can be increased by manipulating the size of the platform and its angle [27].

The results of this study confirm the data originally identified in controlled experimental and/or archaeological assemblages. Most importantly our experiments confirm the importance of platform variables in their effect on the size of lithic products. While platform depth undoubtedly exerts a great influence on blank size and to a large extent on weight, the role of EPA is more significant for 2-dimensional measures of size (i.e. surface area and edge length). Simultaneously increasing EPA and decreasing the thickness of the platform depth will produce blanks with longer edges per unit of mass. Blank morphology, however, is not related to platform variables in a straightforward way, most probably as a result of variables not controlled during production of our experimental assemblage and non-measurable variables that influence blank shape, such as angle of blow [98]. Moreover, effect of EPA on blank size and shape that has been confirmed in controlled experimental conditions was not as strong in our freehand experiment. We believe that the cause probably lies in ways EPA is measured in lithic assemblages. Furthermore, while most of the experiments in controlled settings [70] have not investigated the effect of platform shape on blank shape, as platform width proportionally increases with platform thickness, our dataset shows that managing platform size has an impact on the final blank morphology. As platform width is, to a large extent, determined by core size (namely core width) it can be hypothesized that core morphology is a factor that influences blank shape. Core reduction strategy is thought to have direct effect on blank shape ([3,4] but see [70]). This study, nevertheless, does not take into account the technology used to produce blanks as the production process was not following a particular core reduction systems, rather it relied on basic shape features of blanks. How much platform size, platform width to thickness ratio and EPA reflect morphology of the core or are manipulated immediately before detaching a blank without core morphology playing significant role in blank size/shape, is a question that is beyond the scope of this paper. These results offer an independent confirmation that platform variables offer an insight into size and morphology of the blank that can further be used for estimating properties of unretouched or incomplete elements, the tendencies of blank production process, while in retouched elements it offers an approximate and relative measure of weight and area loss. Combined with other lines of evidence in lithic assemblages, this adds to our understanding of decisions that are made during the blank production process. This also provides some indication of the inferences of behavior that can be investigated when interpreting past behaviors and adaptations. Furthermore, this facet adds to an understanding of the character and organization of lithic technological systems and questions on the evolution of lithic technology.
